# Effect of canola oil consumption on memory, synapse and neuropathology in the triple transgenic mouse model of Alzheimer’s disease

**DOI:** 10.1038/s41598-017-17373-3

**Published:** 2017-12-07

**Authors:** Elisabetta Lauretti, Domenico Praticò

**Affiliations:** Alzheimer’s Center at Temple, Department of Pharmacology Lewis Katz School of Medicine, Temple University, Philadelphia, PA 19140 USA

## Abstract

In recent years consumption of canola oil has increased due to lower cost compared with olive oil and the perception that it shares its health benefits. However, no data are available on the effect of canola oil intake on Alzheimer’s disease (AD) pathogenesis. Herein, we investigated the effect of chronic daily consumption of canola oil on the phenotype of a mouse model of AD that develops both plaques and tangles (3xTg). To this end mice received either regular chow or a chow diet supplemented with canola oil for 6 months. At this time point we found that chronic exposure to the canola-rich diet resulted in a significant increase in body weight and impairments in their working memory together with decrease levels of post-synaptic density protein-95, a marker of synaptic integrity, and an increase in the ratio of insoluble Aβ 42/40. No significant changes were observed in tau phosphorylation and neuroinflammation. Taken together, our findings do not support a beneficial effect of chronic canola oil consumption on two important aspects of AD pathophysiology which includes memory impairments as well as synaptic integrity. While more studies are needed, our data do not justify the current trend aimed at replacing olive oil with canola oil.

## Introduction

Epidemiological and clinical studies have consistently indicated that higher adherence to the Mediterranean diet is associated with a reduced risk of developing mild cognitive impairment and AD, and a reduced risk of progressing from mild cognitive impairment to AD^1,2^. Among the different nutrients that characterize the Mediterranean diet, a lot of attention has been focused on the daily intake of olive oil as the main source of added fat, which is estimated to be quite high in the Mediterranean area populations compared with other geographical regions around the world^3,4^.

There is no doubt that promoting olive oil consumption in non-Mediterranean populations could be a difficult task in part for the fact that this ingredient is generally more expensive than other cooking oils. Rapeseed oil (known also as canola oil) has emerged as a potential substitute for olive oil since has a similar monounsaturated fatty acids content to that of olive oil and an overall favorable fatty acid profile^5^. As result, in recent years the consumption of canola oil has significantly increased in non-Mediterranean countries, due to the lower cost compared with olive oil and the perception that it shares the health benefits of the olive oil^6,7^.

Today canola oil is the third largest vegetable oil by volume after palm and soybean oil worldwide, and in the United States is one of the most widely used oil for human consumption second only to soybean oil^8^. While many studies have investigated the relationship between olive oil with disease incidence, mortality and biomarkers for diseases, studies with canola oil are mainly focused only to biomarkers. Limited and not conclusive scientific evidence would suggest some benefit for canola oil consumption, but results from studies implementing diets containing canola oil in experimental animal models have provided us with conflicting data^9,10^. On the other hand, studies have consistently demonstrated a beneficial effect of olive oil in different mouse models of neurodegeneration^11–13^. A diet rich in olive oil reduced parenchymal and vascular Aβ levels in a model of brain amyloidosis, TgSwDI^14^, and Aβ and tau neuropthaology together with an and improvement of behavioral deficits in the triple transgenic mice (3xTg)^15^. By contrast, no data are available on the effect that canola oil consumption may have on any of these models and the development of their phenotypes. For this reason, in the present paper we assessed the biological effect of chronic administration of a canola oil-rich diet on the 3xTg mice, which are known to develop both amyloid plaques and neurofibrillary tangles^16^.

## Results

### Effect of canola oil on animal body weight

At the beginning of the study, mice randomized to regular diet or canola rich diet did not differ in terms of total body weight: 27.49 ± 0.88 gr (Control); 27.66 ± 1.12 gr (Canola oil). By the end of the study, when the mice were 12 months old, the group on the regular chow had an average body weight of 31.88 ± 0.91 gr, whereas the one receiving canola oil had a significantly much higher weight of 37.71 ± 1.24 gr (p < 0.01) (Supplemental Fig. 1).

### Influence of canola oil-rich diet on behavior in 3xTg mice

**Figure 1:**
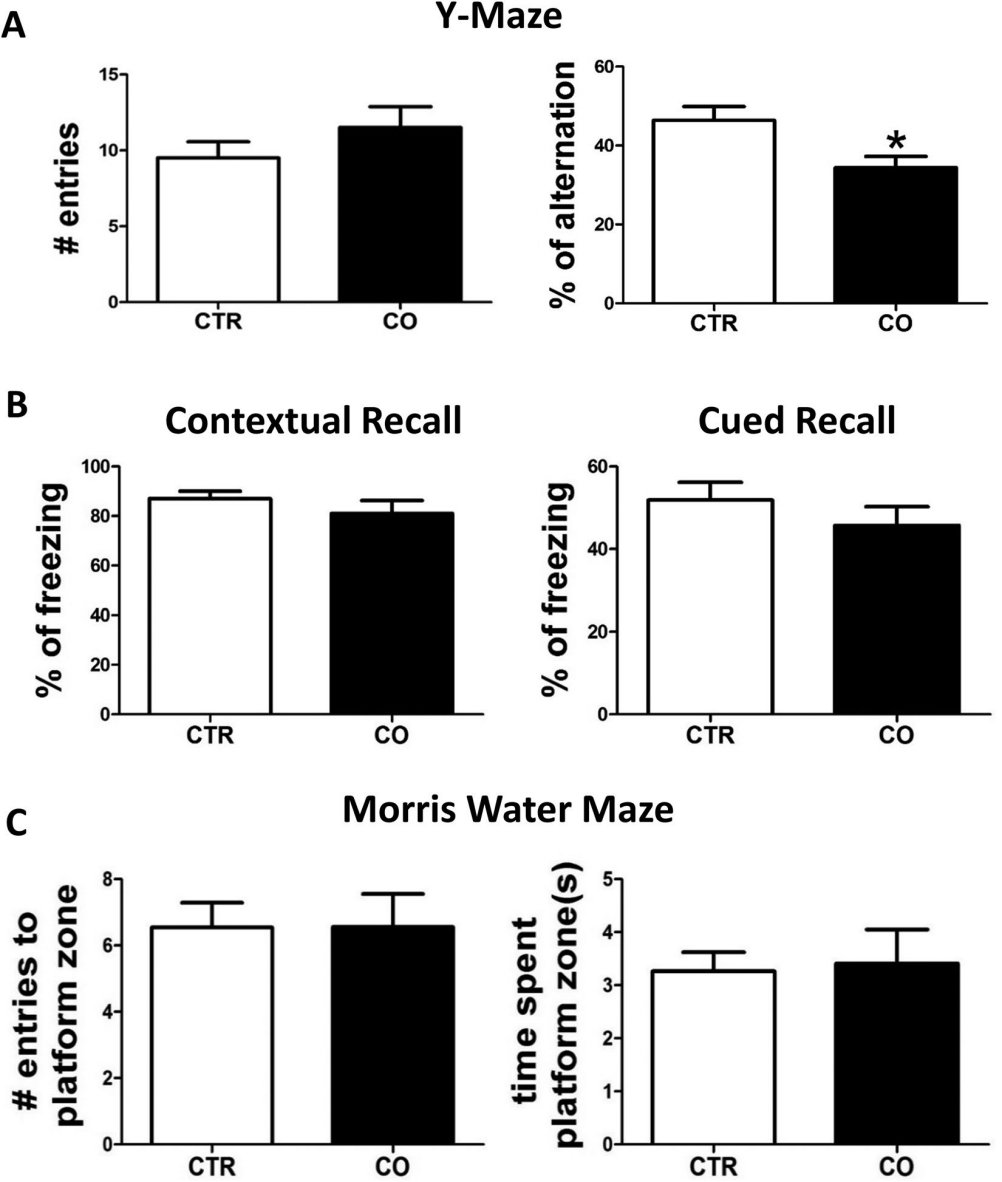
Chronic administration of canola oil-rich diet modulates behavioral responses of 3xTg mice. Six-month old 3xTg mice were randomized to receive regular chow diet (CTR) or diet enriched with canola oil (CO) until they were 12-month-old. (**A**) Mice were tested in the Y-maze paradigm for the number of entries, and the percentage of alternation. (**B**) Percentage of freezing in the contextual and cued phases of the fear conditioning paradigm. (**C**) Mice were also assessed in the Morris water maze paradigm for the number of entries to the platform zone, and the time spent in the platform zone. (CTR n = 11, CO, n = 10) (*p < 0.05).

To determine the effect of canola oil-rich diet on behavior, at the end of the treatment animals were tested in 3 different paradigms: Y-maze, fear conditioning and Morris water maze. As shown in Fig. 1A, in the Y-maze, compared with control group mice receiving the canola rich diet showed a small increase in the number of entries that did not reach statistical significance. By contrast, when we assessed the percentage of alternation, we found that chronic canola oil treatment resulted in statistically significant lower percentage in this parameter when compared with control mice (Fig. 1A).

Next, mice underwent fear conditioning testing. No significant differences between the two groups were observed in the freezing time during the training session (not shown). However, while no changes between the two groups were noted in the contextual recall phase, a trend toward reduction in freezing time was observed for the canola oil group in the cued recall phase of this paradigm (Fig. 1B).

Finally, animals were tested in the Morris water-maze paradigm. All mice in each group were able to reach the training criterion within 4 days and no differences were found during the training session (not shown). During the probe test we observed that compared with controls, mice receiving the canola oil did not manifest any significant difference for the number of entries in the platform zone and time spent in the platform zone (Fig. 1C). No significant differences were observed in both paradigms when males and females were analyzed separately.

### Effect of canola oil supplementation on brain amyloidosis

**Figure 2:**
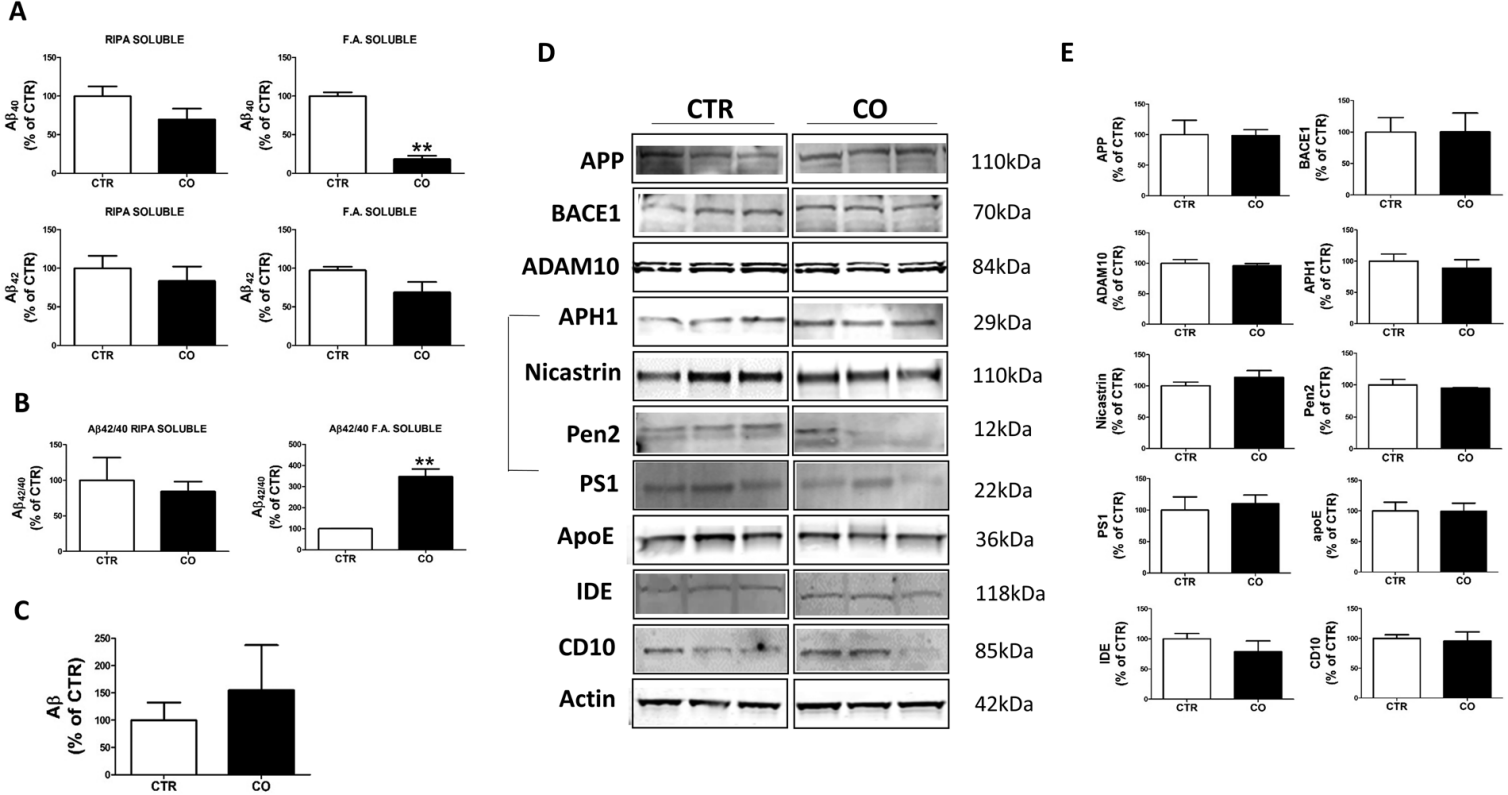
Effect of chronic administration of canola oil-rich diet on brain Aβ levels and deposition. (**A**) RIPA-soluble (RIPA) and formic acid extractable (F.A.) Aβ 1-40 and Aβ 1-42 levels in brain cortex homogenates of 3xTg receiving vehicle (CTR) (n = 5) or canola oil (CO) (n = 5). (**B**) Ratios of Aβ 42/40 for RIPA soluble and formic acid soluble fraction measured in brain from 3xTg controls (CTR) or 3xTg treated with canola oil-rich diet (CO). (**C**) Quantification of the area occupied by Aβ immunoreactivity in brains of 3xTg mice receiving vehicle (CTR) (n = 3) or canola oil (CO) (n = 3). (**D**) Representative Western blots of APP, BACE1, ADAM10, APH1, Nicastrin, Pen2, PS1, ApoE, IDE and CD10 in the brain cortex homogenates from 3xTg mice receiving vehicle (CTR) (n = 4) or canola oil (CO) (n = 4). (**E**) Densitometric analyses of the immunoreactivities to the antibodies shown in the previous panel.

At 12 months of age, mice were euthanized and brain cortex homogenates assayed for Aβ levels in the RIPA-soluble and formic acid-soluble fractions. Compared with controls, we found that canola oil group did not manifest any significant differences in the levels of RIPA-soluble Aβ 1-40 and Aβ 1-42, and that this was also true for the formic acid- soluble fraction of the Aβ 1-42 (Fig. 2A). However, canola oil treated mice had a significant reduction in the formic acid-soluble fraction of the Aβ 1-40 levels (Fig. 2A). An analysis of the Aβ 42/40 ratios for the RIPA-soluble fractions did not reveal any changes between the treated and control mice (Fig. 2B). By contrast, compared with control group mice receiving the canola oil-rich diet had a significant increase for the ratios of the Aβ 42/40 in the formic acid-soluble fraction (Fig. 2B). Next, we investigated the effect of canola oil on Aβ deposition by immunohistochemistry. While no statistically significant differences were observed between the two groups, we noticed that compared with controls canola oil-treated mice had a trend toward an increase in the immunoreactivity for Aβ deposits, which did not reach statistical significance (Fig. 2C).

To investigate possible effect of the active treatment on APP metabolism next we assayed the levels of the Aβ precursor protein (APP) and the proteases involved in its cleavage by Western blot. Compared with controls, no changes in the levels of all proteins investigated were observed when the canola oil group was compared with control mice (i.e., APP, BACE1, ADAM10, Nicastrin, Pen 2, PS1, APH1) (Fig. 2D,E). Finally, we looked at some of the proteins that have been involved in Aβ clearance, but no significant differences were found between the two groups in the levels of apolipoprotein E (APOE), neprilysin (CD10) and insulin degrading enzyme (IDE) (Fig. 2D,E). No differences were observed in any of the parameters above described when males and females were analyzed separately.

### Effect of canola oil rich-diet on tau phosphorylation

**Figure 3:**
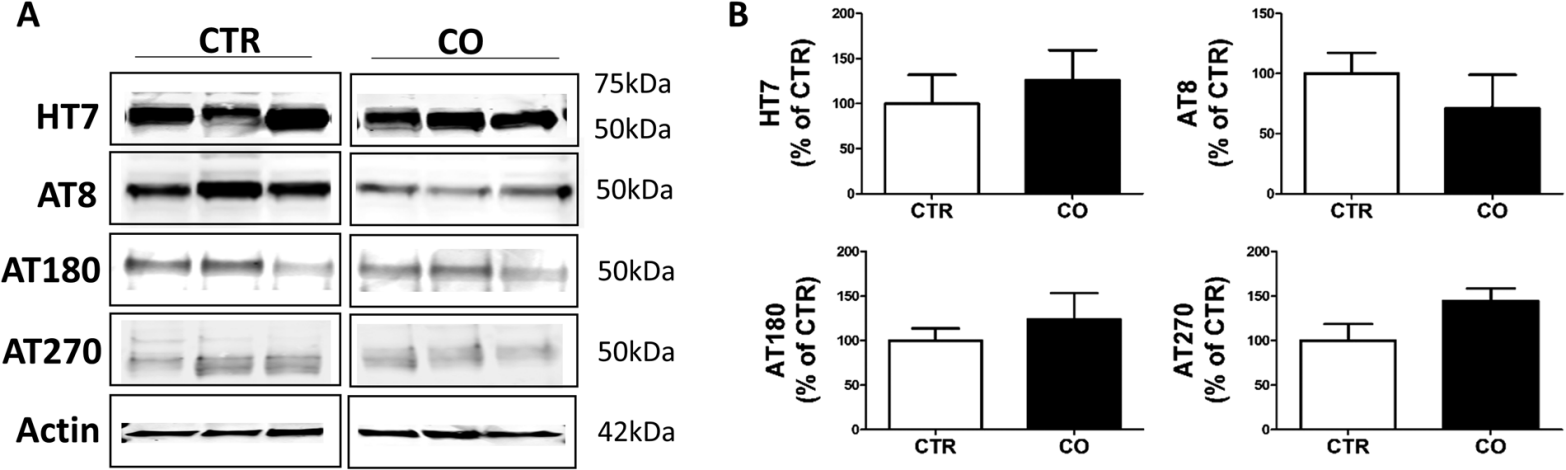
Effect of chronic administration of canola oil-rich diet on tau phosphorylation. (**A**) Representative Western blots of total soluble tau (HT7), phosphorylated tau at residues ser202/thr205 (AT8), thr231/ser235 (AT180), and thr181 (AT270) in brain cortex homogenates from 3xTg mice receiving vehicle (CTR) or canola oil for 6 months. (**B**) Densitometric analyses of the immunoreactivities to the antibodies shown in the previous panel (CO, n = 4; CTR, n = 4).

Next, we investigated the effect of chronic canola oil-rich diet consumption on tau protein levels and phosphorylation in the brain cortices of the same mice. Compared with controls, no significant effect of the treatment was observed when the levels of total tau were assessed (Fig. 3A,B). In a similar manner, no changes were also observed between the two groups of mice when we assessed the levels of tau phosphorylated at the following epitopes: ser202/thr205, t231/s235, and thr181 as recognized by the antibodies AT8, AT180 and AT270 respectively (Fig. 3A,B).

### Effect of canola oil-rich diet on synaptic proteins and neuroinflammation

**Figure 4:**
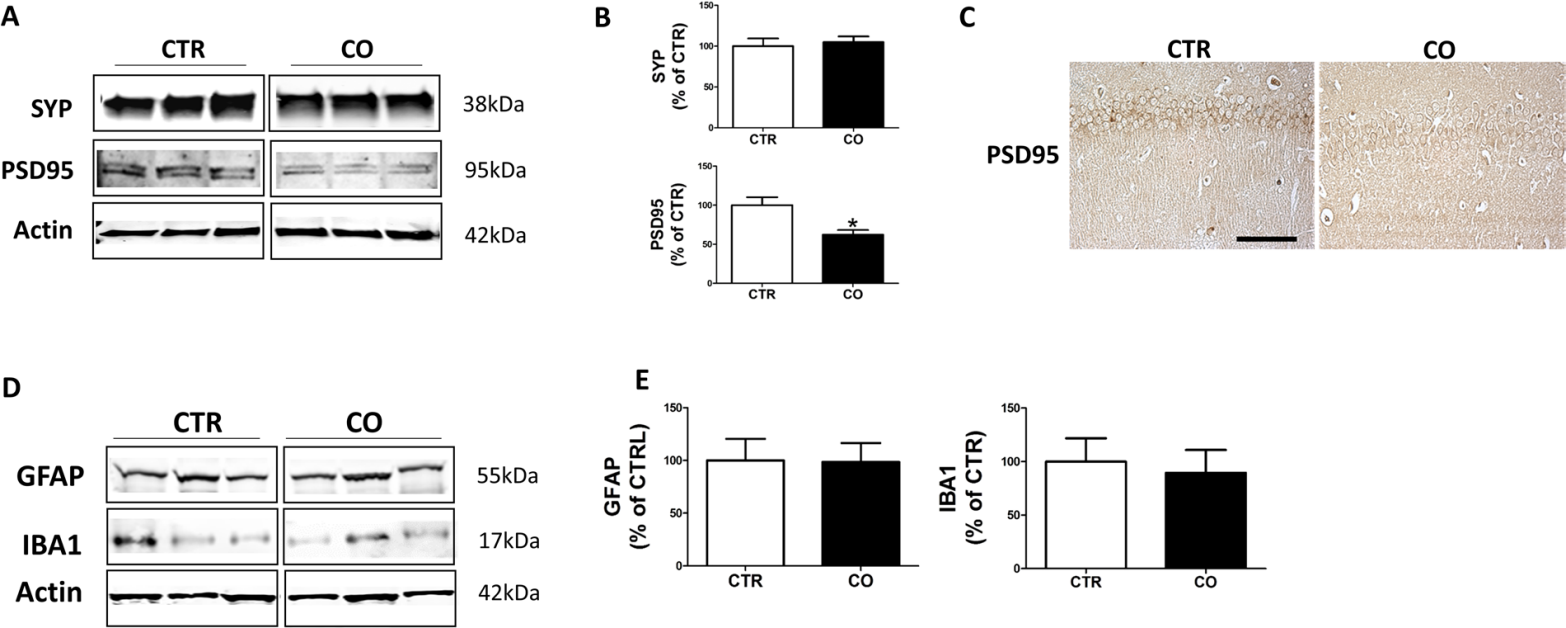
Effect of chronic administration of canola oil-rich diet on synaptic integrity and neuroinflammation. (**A**) Representative western blot analyses of synaptophysin (SYP) and post-synaptic density protein 95 (PSD95) in brain cortex homogenates of 3xTg mice treated with vehicle (CTR) or canola oil (CO). (**B**) Densitometric analyses of the immunoreactivities to the antibodies shown in the previous panel (CTR, n = 4; CO, n = 4) (*p < 0.05). (**C**) Representative images of brain sections from 3xTg mice receiving canola oil (CO) vehicle (CTR) immunostained with PSD95 antibody. (**D**) Representative western blot analyses of GFAP and IBA1 in brain cortex homogenates of 3xTg mice treated with vehicle (CTR) or canola oil (CO). (**E**) Densitometric analyses of the immunoreactivities to the antibodies shown in the previous panel (CTR, n = 4; CO, n = 4).

To assess whether the effect on the behavior response we observed in the canola oil-treated mice was also biochemically characterized by a change in synaptic integrity, we assayed the levels of two major synaptic proteins: synaptophysin (SYP) indices of pre-synaptic integrity, and the postsynaptic density protein 95 (PSD95). As shown in Fig. 4, no differences were observed between the two groups when SYP levels were measured. By contrast, mice fed with canola oil-rich diet when compared with the control group displayed a statistically significant decrease in the levels of PSD95 protein (Fig. 4A,B). Confirming the immunoblot data, canola-treated mice showed a significant decrease in brain immunoreactivity for this synaptic protein (Fig. 4C).

Next we assessed whether two well-established markers of neuroinflammation were influenced by the dietary regimen. Compared with controls, mice receiving the canola oil-rich diet did not show any significant differences in the levels of GFAP, a marker of astrocytes activation, and IBA1, a marker of microglia activation (Fig. 4D,E). No significant differences were observed in any of the parameters above described when males and females were analyzed separately.

### Effect of canola oil-rich diet on CREB signaling on 3xTg mice

**Figure 5:**
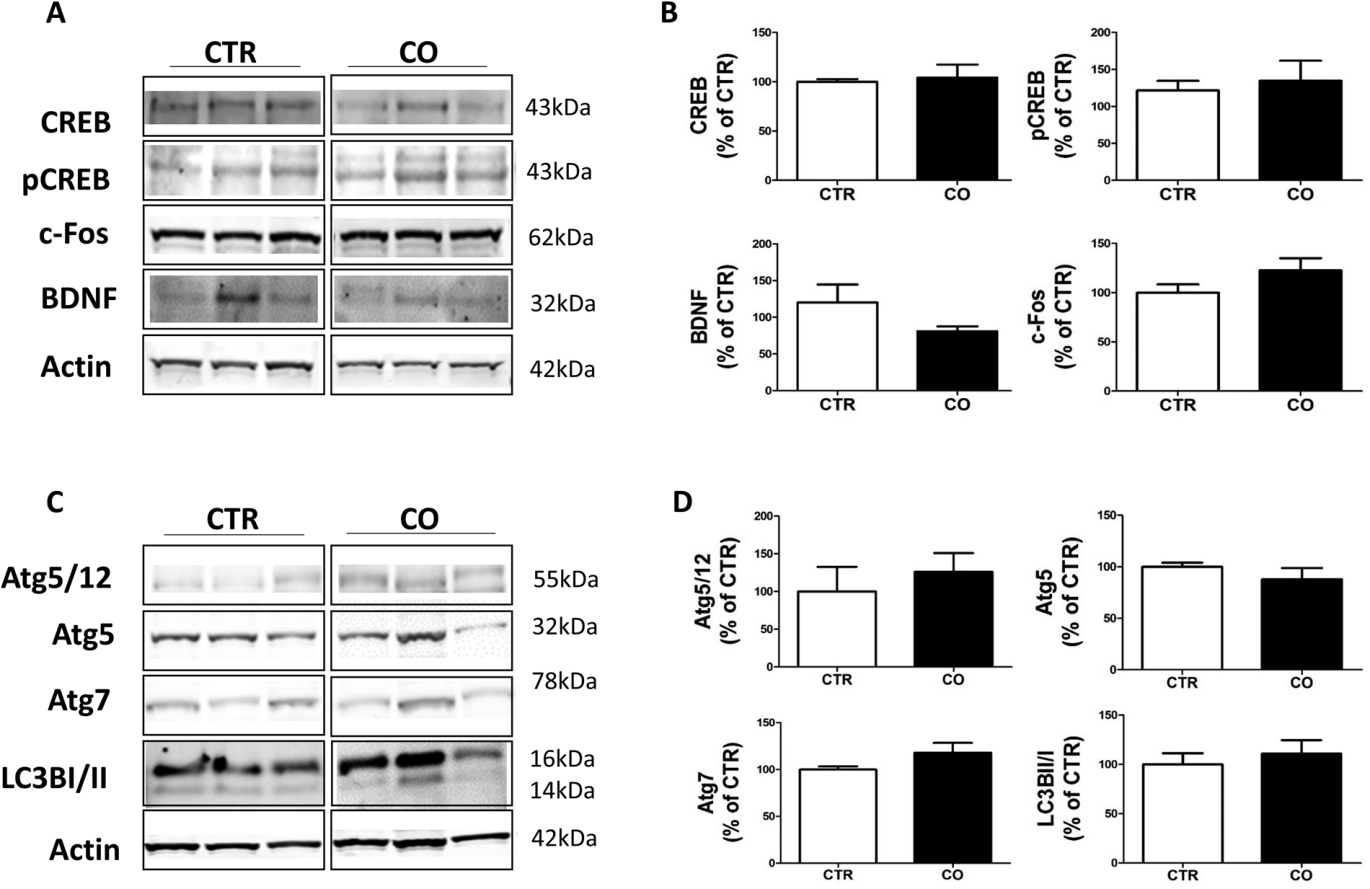
Effect of chronic administration of canola oil-rich diet on CREB signaling and autophagy. (**A**) Representative Western blot analyses of CREB pCREB, c-Fos, BDNF, in brain cortex homogenates of 3xTg mice receiving vehicle (CTR) or canola oil (CO) for 6 months. (**B**) Densitometric analyses of the immunoreactivities to the antibodies shown in the previous panel (CTR, n = 3, CO, n = 3). (**C**) Representative Western blot analyses of Atg5/12, Atg5, Atg7, LC3BI/II in brain cortex homogenates of 3xTg mice receiving vehicle (CTR) or canola oil (CO). (**D**) Densitometric analyses of the immunoreactivities to the antibodies shown in the previous panel (CTR, n = 4, CO, n = 4).

Since CREB and CREB-regulated proteins have been previously reported to be altered in AD pathology, next we investigated the effect of the canola oil-rich diet on total CREB levels and its phosphorylated form at Ser133 (pCREB). As shown in Fig. 5, the levels of total CREB and pCREB were not changed in the brains of canola oil-treated mice compared to controls. Additionally, no differences between the two groups were detected for the expression levels of c-Fos and brain derived neutrophic factor (BDNF), two important CREB target genes (Fig. 5A,B).

### Effect of canola oil-rich diet on autophagy in 3xTg mice

Finally, since other vegetable oils such as olive oil have been reported to influence autophagy markers, next we wanted to assess whether this was also the case for canola oil. Among the autophagy markres, we assessed autophagy protein 5 (Agt5), Atg5/12, Atg7 and the microtubule-associated protein light chain 3 conversion (LC3BI/II) which are considered essential for the autophagosome formation and autophagic flux, respectively^17,18^. Compared with the control group, mice receiving the canola oil-rich diet did not manifest any significant differences in the steady state levels of Agt5, Atg5/12, Atg7, as well as LC3BI/II conversion ratio (Fig. 5C,D).

## Discussion

The data presented in the current paper demonstrate that chronic administration of a diet enriched with canola oil results in significant deficits of working memory and synaptic pathology, but has no effect on the Aβ deposits and tau phosphorylation levels in a transgenic Alzheimer’s mouse model that develops Aβ deposits and tau neurofibrillary tangles.

Increasing evidence has been accumulated showing that nutritional factors can influence diverse aspects of general health by modulating specific biological systems^19^. Thus, over the past two decades substantial research has recognized that chronic exposure to the Mediterranean diet is beneficial with respect to reducing the incidence of cardiovascular diseases, and metabolic syndrome^20^. In addition, longitudinal and prospective clinical trials have revealed that higher adherence to this type of diet is associated with slower rates of cognitive decline, reduced conversion to AD, and improvement of cognitive function^21,22^. Among the key elements of the Mediterranean diet, an important role has been attributed to daily intake of fresh fruits and vegetables, and the usage of olive oil as a primary source of fat^23^. In particular, regular daily olive oil consumption has been suggested as the most important and integral component of the diet, and as having a major role in the health benefit of this diet^24,25^.

This concept has been the propeller for some health organizations in non-Mediterranean countries to promote a Mediterranean diet and the usage of olive oil as the main source of dietary fat. However, this policy has not always been very successful since adopting this type of oil could be more expensive in comparison with other cooking oils in these populations. For this reason, in recent years these countries have been looking for potential alternative to the olive oil. Among them, canola oil has gained increasing attention as a suitable substitute to olive oil especially in countries that lack the primary source for it: the olive tree. As result, canola oil consumption is now quite high in many of these countries because of its lower price compared with olive oil, but also and most importantly because there is a diffuse perception that the canola oil is a healthy choice.

Most of the studies so far investigating the relationship between canola oil consumption and health benefits have shown limited evidence of beneficial effects or neutral action on biomarkers of risk factors for cardiovascular diseases^26^. On the other hand, studies have provided conflicting results depending on the experimental model implemented, the length of the treatment and the particular end-point considered^27,28^. However, no data are available on the biological effects that chronic exposure to dietary canola oil may have on cognitive function and the development of the AD-like phenotype which typically include: memory, synaptic integrity, Aβ and tau neuropathology.

To address this scientific question, we implemented a dietary approach and utilized a transgenic mouse model, the 3xTg mice, which manifest all these aspects including memory impairments, Aβ deposits and tau tangles pathology^16^.

First, we observed that compared with 3xTg mice receiving regular chow diet, the group treated with canola oil-rich diet had a significant increase in body weight suggesting that the added oil provided extra calories to the mice. This observation is in contrast with previous reports showing that chronic diet supplementation with canola oil had no effect on the average animal body weight^28,29^. We interpret this discrepancy as secondary to the different strains of mice that were implemented in those studies, and probably the length of our study.

However, this fact did not translate in any alteration of their motor ability since we did not observe any differences between the two groups when for instance the animals were tested in the different behavioral paradigms. Thus, in the Y-maze no significant differences were observed between the two groups when the number of entries in each arm of the maze was considered suggesting that the diet and the higher body weight did not alter the motor ability of the mice. By contrast, compared with 3xTg kept on a regular chow diet, the ones receiving canola oil-supplemented diet had a significant reduction in the percentage of spontaneous alternations in the Y-maze, suggesting an impairment of their working memory^30^.

Supporting the detrimental effect of chronic exposure to canola oil-rich diet on the behavior responses, we found that the same mice had biochemical evidence for a reduction in synaptic integrity as demonstrated by the significantly lower levels of PSD95 protein, a well-established synaptic marker, in the brains of the canola oil-treated mice^31^.

Analysis of the amount of Aβ 1-40 and Aβ 1-42 peptides in the soluble fractions from brain cortices of these mice did not show any significant differences between the two groups. A similar result was obtained when we assayed the formic acid soluble fraction of the Aβ 1-42 peptides. By contrast, we observed that brain samples from mice treated with the canola oil had a significant reduction the formic acid soluble fraction of Aβ 1-40 peptides, which is considered less prone to precipitate and form insoluble deposits compared to the Aβ 1-42 peptides^32^. Normally Aβ 1-40 is produced at higher levels, but as Aβ 1-42 is more hydrophobic and has a stronger tendency to polymerize into neurotoxic species, it seems to be of particular importance in AD pathogenesis^33,34^. This is supported by studies on mutations in APP, presenilin 1 (PSEN1) and PSEN2, which show an increased Aβ 42/40 ratio^35^. Interestingly, an analysis of the ratios among the two fractions of Aβ peptides revealed that the brains of the mice receiving canola oil had a statistically significant increase in the ratios of Aβ 42/40 suggesting a shift towards the more prone to fibril formation and insoluble form of these peptides, which would favor their progressive precipitation and intracellular accumulation.

This observation has great biologic importance, since data in the literature have shown that from a mechanistic point of view elevation in Aβ 42/40 peptide ratio enhances the nucleation and fibrillogenesis of pathogenic Aβ 1-42 peptides, events that are otherwise compromised by the presence of high levels of secreted Aβ 1-40 peptides^36^. Confirming this aspect of the Aβ peptides neurobiology, we found a trend towards an increase in the amount of Aβ deposits immunoreactivity in the brains of the canola oil-treated mice compared with controls.

No significant effect of the canola oil-rich diet was found on some of the major protein systems in place to control Aβ clearance and degradation. Thus, steady state levels of apoE, a major Aβ chaperone, levels of neprilysin and IDE, two major Aβ catabolic pathways, were no different between the controls and canola oil-treated mice.

Since this model is known to develop high levels of phosphorylated tau protein and ultimately forms neurofibrillary tangles, next we were very interested in assessing whether our dietary treatment had any influence on this aspect of their phenotype. By the end of the chronic treatment, levels of total soluble tau and different phosphorylated isoforms were undistinguishable between the two groups, suggesting that canola oil does not influence tau metabolism.

Since previous works have shown that olive oil has a potent anti-inflammatory action *in vivo*, next we assessed the effect of chronic canola oil exposure on classical biomarkers of activation for microglia and astrocytes, two major cellular components and modulators of neuroinflammatory responses^37^. Brain homogenates from canola oil treated mice were not different from the ones receiving chow diet controls when the steady state levels of GFAP, a marker of astrocytosis, and IBA1, a marker of microglia activation, were measured.

Finally, since we previously reported that olive oil is an activator *in vivo* of the autophagic machinery^15^, we also investigated whether or not this was the case in the mice receiving the canola oil-rich diet. Assessment of several well-established markers of autophagy activation in the brain of the two groups of mice did not show any significant differences, suggesting that canola oil does not influence this system^38^.

In conclusion, our investigation demonstrates for the first time to the best of our knowledge a negative effect of the chronic consumption of canola oil on memory, synaptic integrity and Aβ 42/40 ratios in a mouse model of AD. The translational value of our findings lies in the observation that this type of oil supplementation can influence some of the most important features of the AD pathological phenotype.

Overall our findings do not provide support to some of the current ideas suggesting healthy benefits deriving from the regular consumption of canola oil. Although we recognize that more studies are needed to investigate the biological effects of this oil, our data would not justify the increasing tendency of replacing olive oil with canola oil as part of a good and healthy dietary alternative in non-Mediterranean countries.

## Methods and Materials

### Animals and treatment

The study was approved by the Temple Institutional Animal Care and Usage Committee, in accordance with the US National Institutes of Health guidelines. The 3xTg mice harboring 3 transgenes (PS1 _M146V_, tau _P301L_, and APP _Swe_) were used in this study^16^. For the study we used both male and female mice. Six-month old mice were randomized into two groups: one fed with standard diet (CTR, n = 12), the other with canola oil-enriched diet (50 mg/Kg) (CO, n = 10) for 6 months. The control mice used in this study have been previously described^15^. The source of the canola oil added to the diet was “Mazola®” canola oil, which contains 21% total fat (1gr saturated fatty acids, 4gr polyunsaturated fatty acids, and 8 gr of monounsaturated fatty acids per serving). Fresh diet was provided every other day. Mice underwent behavioral tests at 12 months (6 months treatment) of age as described below and a week later euthanized. After perfusion, brains were removed and immediately dissected in two halves: one was stored at −80 °C for biochemistry; the other fixed in 4% paraformaldehyde in phosphate-buffered saline, pH7.4 for immunohistochemistry.

### Behavioral tests

All the animals were handled for at least 3–4 consecutive days before testing, and tests were performed in a blind fashion without knowledge of treatment by the investigator.

### Y-maze

The Y-maze apparatus consisted of 3 arms, 32 cm (long) 610 cm (wide) with 26-cm walls (San Diego Instruments, San Diego, CA). The test was performed as previously described in details in our publications^39,40^.

### Fear conditioning

The fear conditioning test was performed in a conditioning chamber (19 × 25 × 19 cm) equipped with black methacrylate walls, transparent front door, a speaker, and grid floor (Start Fear System; Harvard Apparatus) as previously described in our publications^39^.

### Morris water maze

The test was performed following our published protocol which uses circular pool filled with water maintained at 22° +/−2 °C, and made opaque by the addition of a nontoxic white paint. Mice were given four daily trials for four consecutive days, and on the fifth day a probe trial was administered as previously described in details in our papers^39–41^.

### Immunoblot analyses

Immunoblot analyses were performed as previously described^39–41^. Briefly, proteins were extracted in enzyme immunoassay buffer containing 250 mM Tris base, 750 mM NaCl, 5% NP-40, 25 mM EDTA, 2.5% sodium deoxycholate, 0.5% sodium dodecyl sulfate and an EDTA-free protease and phosphatase inhibitors cocktail tablet (Roche Applied Science, Indianapolis, IN, USA), sonicated, centrifuged at 45 000 r.p.m. for 45 min at 4 °C, and supernatants used for immunoblot analysis, as previously described^39–41^.

### Biochemical analysis

Brain homogenates were sequentially extracted first in RIPA buffer and then in formic acid for the measurement of Aβ 1-40 and Aβ 1-42 peptide levels as previously described^39–42^.

### Immunohistochemistry

Primary antibodies used in this study are listed in Table 1. Immunostaining was performed using serial 6-μm thick coronal sections, as reported previously in details^39–42^. Consecutives sections were incubated in the absence of primary antibodies to ensure specificity of staining.

**Table 1:** Antibodies used in the study.

Antibody	Immunogen	Host	Application	Source	Catalog Number
4G8	aa 18-22 of human beta amyloid (VFFAE)	Mouse	IHC	Covance	SIG-39220
APP	aa 66-81 of APP {N-terminus}	Mouse	WB	Millipore	MAB348
BACE1	aa human BACE (CLRQQHDDFADDISLLK)	Rabbit	WB	IBL	18711
ADAM10	aa 732-748 of human ADAM 10	Rabbit	WB	Millipore	AB19026
PS1	aa around valine 293 of human presenilin 1	Rabbit	WB	Cell Signaling	3622 S
Nicastrin	aa carboxy-terminus of human Nicastrin	Rabbit	WB	Cell Signaling	3632
APH1	Synthetic peptide from hAPH-1a	Rabbit	WB	Millipore	AB9214
Pen2	aa N-terminal of human and mouse Pen-2	Rabbit	WB	Invitrogen	36-7100
HT7	aa 159-163 of human tau	Mouse	WB	Thermo	MN1000
AT8	Peptide containing phospho-S202/T205	Mouse	WB	Thermo	MN1020
AT180	Peptide containing phospho-T231/S235	Mouse	WB	Thermo	P10636
AT270	Peptide containing phospho-T181	Mouse	WB	Thermo Scientific	MN1050
PSD95	Purified recombinant rat PSD-95	Mouse	WB, IHC	Thermo	MA1-045
SYP	aa 221-313 of SYP of human origin	Mouse	WB	Santa Cruz	sc-55507
IBA1	Linear peptide corresponding to human IBA1	Mouse	WB	Millipore	MABN92
GFAP	spinal chord homogenate of bovine origin	Mouse	WB	Santa Cruz	sc-33673
Atg5/12	KLH-conjugated linear peptide corresponding to human ATG5	Rabbit	WB	Millipore	ABC14
Atg7	Synthetic peptide corresponding to N-term of ATG7	Rabbit	WB	Cell Signaling	2631
LC3BI/II	Synthetic peptide corresponding to N-term of LC3B	Rabbit	WB	Cell Signaling	2775
CD10	aa 230-550 mapping within an internal region of CD10 of human origin	Rabbit	WB	Santa Cruz	sc-9149
IDE	Synthetic peptide corresponding to N-term of human IDE	Goat	WB	Santa Cruz	sc-27265
ApoE	19-311 mapping at the C-terminus of apoE of mouse origin	Rabbit	WB	Santa Cruz	sc-98574
CREB	Synthetic peptide corresponding to N-term of human CREB	Rabbit	WB	Cell Signaling	9197
pCREB	Synthetic peptide corresponding to the residues surrounding Ser133 of CREB	Rabbit	WB	Cell Signaling	9198
c-Fos	Peptide mapping the internal region of human c-Fos	Rabbit	WB	Santa Cruz	sc-253
BDNF	Peptide mapping the internal region of human BDNF	Rabbit	WB	Santa Cruz	sc-456
Actin	gizzard Actin of avian origin	Mouse	WB	Santa Cruz	sc-47778

WB: Western blot; IHC: immunohistochemistry.

### Data analysis

Unpaired Student’s *t*-test (two-sided) and one-way ANOVA were performed using Prism 5.0 (GraphPad Software, La Jolla, CA, USA). All data are presented as mean ± s.e.m. Significance was set at *P* < 0.05.

### Availability of data and materials

The datasets generated and analyzed during the current study are available from the corresponding author on reasonable request.

## Electronic supplementary material

supplemental information
