# Supplementary material for: Effect of canola oil consumption on memory, synapse and neuropathology in the triple transgenic mouse model of Alzheimer’s disease
Source: Sci Rep. 2017 Dec 7;7:17134. doi: 10.1038/s41598-017-17373-3 (PMC5719422; doi:10.1038/s41598-017-17373-3)

Effect of canola oil consumption on memory, synapse and neuropathology in the triple transgenic model of Alzheimer's disease

Elisabetta Lauretti, Domenico Praticò

Department of Pharmacology and Center for Translational Medicine, Lewis Katz School of Medicine, Temple University, Philadelphia, PA 19140.

Correspondence and requests for materials should be addressed to: Domenico Praticò, M.D. 947, Medical Education and Research Building 3500 North Broad Street Philadelphia, PA 19140 E-mail: praticod@temple.edu.

Supplementary information

Supplementary figure 1: Effect of canola oil on body animal weight. Starting at 6 months of age 3xTg mice were randomized to receive regular chow (CTR) or chow enriched with canola oil (CO). Their body weight was assessed at the beginning and each month thereafter until the end of the study. Animal weight of 3xTg mice fed with regular chow diet (CTR, n=12) and canola oil-rich diet (CO, n=10) (*p<0.01).

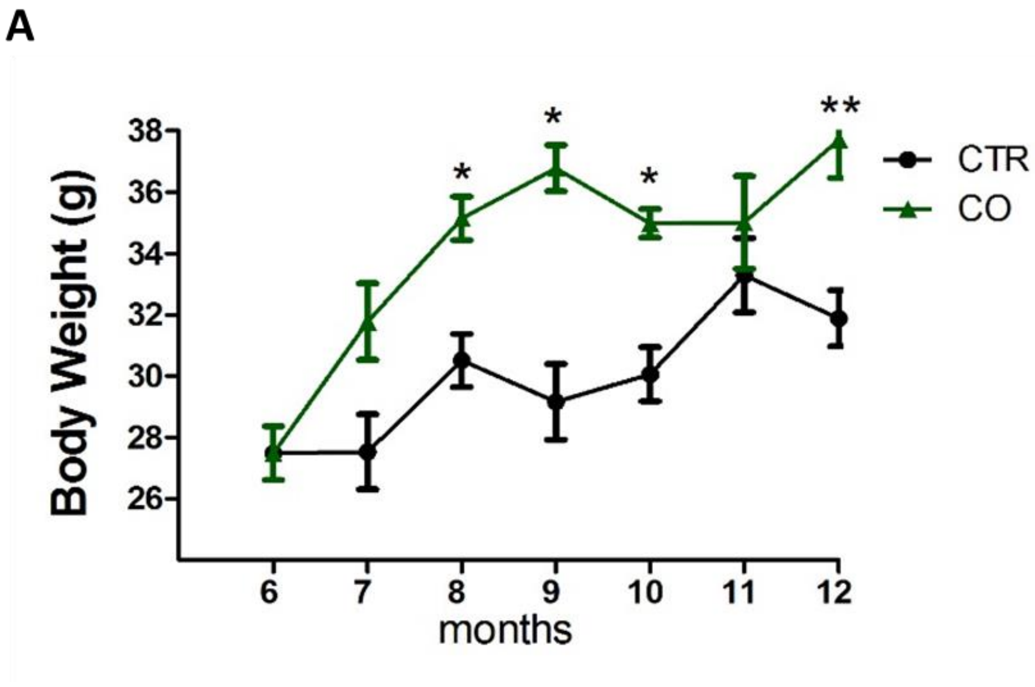

Supplementary Figure 2. Full length western blots used within the article. Throughout this article, blots are cropped to improve conciseness of the presentation. Where images of blots are cropped, a minimum of at least three band is shown. Representative examples of bands of interest displayed in the corresponding figures are shown here in their entirety. The molecular weight standard is shown on the left of each blot (in kDa).

Fig 2. Nicastrin (110-120kDa)

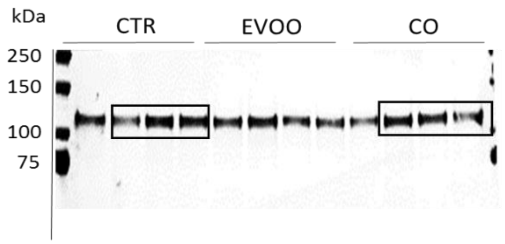

Fig 2. PS1 (22kDa)

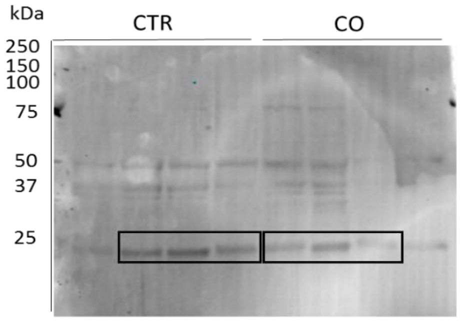

Fig 3. AT270 (75-50kDa)

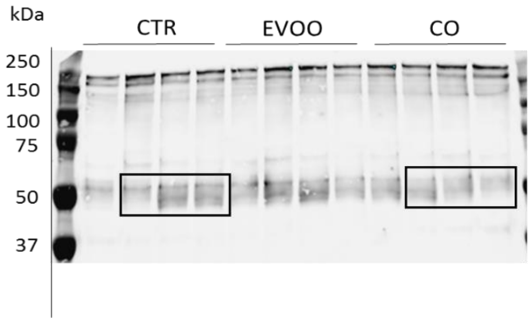

Fig 3. HT7 (75-50kDa)

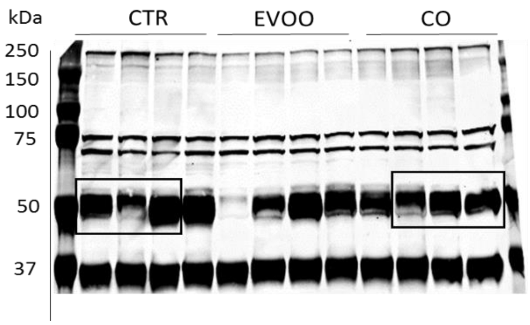

Fig 4. GFAP (50-48kDa)

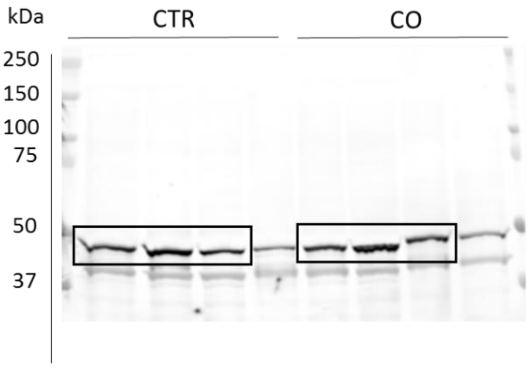

Fig 5. LC3BI/II (16-14kDa)

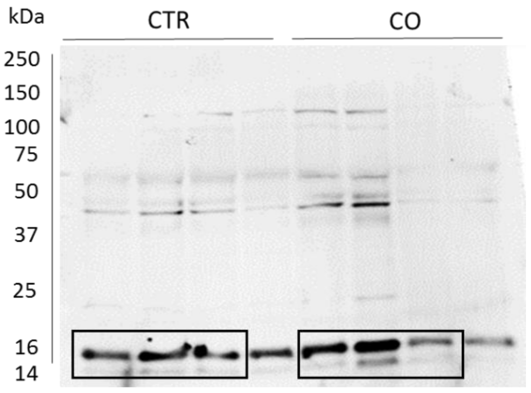

Supplement: Supplementary file 1 — supplemental information [file 41598_2017_17373_MOESM1_ESM.pdf]
